# Involvement of Pro-Inflammatory Macrophages in Liver Pathology of Pirital Virus-Infected Syrian Hamsters

**DOI:** 10.3390/v10050232

**Published:** 2018-05-02

**Authors:** Corey L. Campbell, Aaron T. Phillips, Amber Rico, Amanda McGuire, Tawfik A. Aboellail, Sandra Quackenbush, Ken E. Olson, Tony Schountz

**Affiliations:** 1 Arthropod-Borne and Infectious Diseases Laboratory, Department of Microbiology, Immunology and Pathology, College of Veterinary Medicine and Biomedical Sciences, Colorado State University, Fort Collins, CO 80523, USA; Corey.Campbell@ColoState.EDU (C.L.C.); aaronphi@uab.edu (A.T.P.); arico3@unl.edu (A.R.); Amanda.Mcguire@colostate.edu (A.M.); Kenneth.Olson@ColoState.EDU (K.E.O.); 2 Department of Microbiology, Immunology and Pathology, College of Veterinary Medicine and Biomedical Sciences, Colorado State University, Fort Collins, CO 80523, USA; Tawfik.Aboellail@ColoState.EDU (T.A.A.); Sandra.Quackenbush@colostate.edu (S.Q.)

**Keywords:** arenavirus pathogenesis, Syrian hamster, transcriptome profiling, hematology, host response

## Abstract

New World arenaviruses cause fatal hemorrhagic disease in South America. Pirital virus (PIRV), a mammarenavirus hosted by Alston’s cotton rat (*Sigmodon alstoni*), causes a disease in Syrian golden hamsters (*Mesocricetus auratus*) (biosafety level-3, BSL-3) that has many pathologic similarities to the South American hemorrhagic fevers (BSL-4) and, thus, is considered among the best small-animal models for human arenavirus disease. Here, we extend in greater detail previously described clinical and pathological findings in Syrian hamsters and provide evidence for a pro-inflammatory macrophage response during PIRV infection. The liver was the principal target organ of the disease, and signs of Kupffer cell involvement were identified in mortally infected hamster histopathology data. Differential expression analysis of liver mRNA revealed signatures of the pro-inflammatory response, hematologic dysregulation, interferon pathway and other host response pathways, including 17 key transcripts that were also reported in two non-human primate (NHP) arenavirus liver-infection models, representing both Old and New World mammarenavirus infections. Although antigen presentation may differ among rodent and NHP species, key hemostatic and innate immune-response components showed expression parallels. Signatures of pro-inflammatory macrophage involvement in PIRV-infected livers included enrichment of *Ifng*, *Nfkb2*, *Stat1*, *Irf1*, *Klf6*, *Il1b*, *Cxcl10*, and *Cxcl11* transcripts. Together, these data indicate that pro-inflammatory macrophage M1 responses likely contribute to the pathogenesis of acute PIRV infection.

## 1. Introduction

Pathogenic mammarenaviruses cause substantial morbidity and mortality each year. It is estimated that more than 300,000 cases with about 5000 deaths are caused by Lassa virus (LASV) in Africa annually [1]. In South America, more than 50,000 cases of hemorrhagic fever, most from Junín virus (JUNV; Argentine hemorrhagic fever) infection, with a fatality rate of about 20%, have occurred since the 1950s [2]. Pathogenic arenaviruses are hosted by peridomestic rodents, thus outbreaks of zoonotic arenavirus disease in humans will continue to be a public health risk in these areas [3]. Arenaviruses that cause severe hemorrhagic disease in humans require biosafety level-4 (BSL-4) containment, which limits the number of institutions where pathogenesis studies can be performed, and few animal models that recapitulate human arenavirus hemorrhagic fevers (AHF) are available. Laboratory strains of house mice (*Mus musculus*) have been used; however, specific gene knock-outs are required, which substantially compromises their value as models for human arenavirus disease [4]. Non-human primates (*Macaca mulatta*, *Macaca fascicularis*) have also been used for Lassa fever models, and these share many features of human disease [5,6]. Jamaican fruit bats (*Artibeus jamaicensis*) infected with Tacaribe virus also develop clinical signs with strong resemblance to human disease [7]. In addition, guinea pigs (*Cavia porcellus*) and Syrian golden hamsters (*Mesocricetus auratus*) have been used in New World arenavirus studies. These two rodent species were found to exhibit many pathologic similarities to human disease and, hence, they are favored small-animal models [8,9].

Pirital virus (PIRV, *Pirital mammarenavirus*) was first isolated from Alston’s cotton rats (*Sigmodon alstoni*) on a farm near Caño Delgadito, Venezuela, in 1997 [10] and were subsequently determined to be a natural reservoir host. PIRV is divergent from other New World arenaviruses; isolates exhibit substantial genetic diversity despite antigenic similarity [10,11,12], and some evidence suggests PIRV has undergone homologous recombination with other viruses [13]. Spillover of Guanarito virus (GTOV), which causes Venezuelan hemorrhagic fever, into Alston’s cotton rats has occurred [14]; therefore, coinfection with PIRV could lead to novel recombinants or reassortants with zoonotic potential.

Experimental infection of Syrian hamsters with PIRV causes a disease with many similarities to human arenavirus disease [15]. Clinical and pathological parallels to Argentine hemorrhagic fever include pulmonary and splenic hemorrhage, focal necrosis of the liver, and reticulum cell hyperplasia. Similarities to Lassa fever are more striking and include leukocytosis, megalothrombocytosis and coagulopathy, liver necrosis, and elevated transaminases. Although no human cases of PIRV disease have been documented, it is considered a BSL-3 agent because of its severe hemorrhagic disease in Syrian hamsters [8,9].

We infected Syrian hamsters with PIRV to extend pathological findings from previous reports [15]. We sought to substantiate the use of hamsters as a model for human arenavirus disease by identifying parallels in tissue pathology and immune pathway transcriptional profiles in PIRV-infected hamsters compared to those reported in arenavirus-infected non-human primate (NHP) studies [16,17]. We expected to find similarities among both Old and New World arenaviruses, due to the divergence of PIRV from strict categorization as a New World virus. Because of the prominent hepatic disease, transcriptional profiling of liver RNA was performed. More than 3400 differentially expressed transcripts were identified, which represented metabolic and host-response pathways. Many transcripts representing hemostasis and antiviral pathways were modulated. Notably, several transcripts expressed by T cells were elevated, including interferon-γ (IFNγ); however, signatures of T-cell exhaustion and Tr1 regulatory T cells were also present, suggesting suppression of the adaptive response. Many parallels with arenavirus-infected NHP were identified, suggesting the Syrian hamster may be a suitable surrogate model for the study of human arenavirus disease.

## 2. Materials and Methods

### 2.1. Ethics Statement

All work was approved by the Colorado State University Animal Care and Use Committee (protocol 14-4864A, 26 January 2016), Biosafety Committee, and in compliance with the USA Animal Welfare Act.

### 2.2. Hamster Infections

Because PIRV causes significant hemorrhagic disease in hamsters but is not known to infect humans, it is manipulated under BSL-3 containment [10]. All infection experiments were performed in BSL-3 laboratories with appropriate personal protective equipment (PPE), including powered, air-purifying respirators (PAPRs) and Tyvek suits. Pirital virus strain VAV-488 stocks were produced on Vero E6 cells (CRL-1586). Hamsters were anesthetized by inhalation of isoflurane and intramuscularly (IM) inoculated with 10^4^ TCID_50_ of PIRV, diluted in sterile phosphate-buffered saline (PBS), into the right hindquarters. Hamsters were monitored twice daily for signs of disease, the appearance of which was the criterion for humane euthanasia. Terminal blood was collected by cardiac puncture into ethylenediaminetetraacetic acid (EDTA) and serum separator tubes (SST) at the time of euthanasia for hematology, and blood chemistry and serology, respectively. Tissues harvested for RNA extraction were placed in cryovials and snap-frozen in an acetone/dry ice bath, whereas tissues for histopathology and immunochemistry were collected in 10% buffered formalin.

### 2.3. Hematology and Blood Chemistry

Three groups were analyzed by one-way analysis of variance (ANOVA); uninfected control (*n* = 3), euthanized due to disease (*n* = 6), and infected (asymptomatic) survivors (*n* = 3). Hematology was performed on whole blood collected in EDTA (Becton–Dickinson, Franklin Lakes, NJ, USA) using a Heska HemaTrue Analyzer. Blood collected into SST tubes (Becton-Dickinson) was centrifuged at 1000× *g* for 10 min at 4 °C and serum analyzed with a Heska DRI-CHEM 4000 chemistry analyzer (Heska, Loveland, CO, USA).

### 2.4. Histopathology and Immunohistochemistry

Paraffin-embedded formalin-fixed tissues were rehydrated, treated with citrate buffer at 90 °C for 15 min, and blocked with SuperBlock T20 (Thermo, Rockford, IL, USA). Anti-PIRV mouse ascites fluid was used at 1:150 dilution and incubated overnight at 4 °C. Unbound primary antibody was removed by washing 3× with Tris-buffered saline containing 0.03% TWEEN-20 (TBST). Endogenous peroxidase was quenched with 0.3% hydrogen peroxide in PBS for 5 min followed by washing 2× in TBST. Secondary antibody was horseradish peroxidase-conjugated anti-mouse IgG (A4416, Sigma, St. Louis, MO, USA) used at a 1:2000 dilution and incubated for 30 min at room temperature. Slides were again washed 3× with TBST. 3,3′-diaminobenzidine (DAB) was added to the slides and allowed to develop chromogen for 5 min. Hematoxylin was used to counterstain. Complete necropsies were performed on all study animals to record gross pathologic lesions and to characterize the nature and severity of histologic lesions for later corroboration with transcriptome data. A pathologist examined the histology slides blindly. A lesion scoring system was devised where: (1) indicates that there were no significant histologic lesions; (2) minimal indicates that <5% of total parenchyma was involved; (3) mild indicates that 5–10% of the parenchyma was involved; (4) moderate where 10–25% of the parenchyma was involved; (5) moderately severe indicates that 25–50% of the parenchyma was involved; and (6) marked indicates that 50–75% or more of the parenchyma was involved.

### 2.5. Enzyme-Linked Immunosorbent Assay (ELISA)

A sequence fragment of PIRV VAV-488 S segment (1717-2457, AF485262) was codon-optimized for *E. coli* expression and de novo synthesized with flanking 5′ *Eco*RI and 3′ *Not*I restriction sites (GeneArts). The fragment was subcloned into pET-23b with vector start and C-terminal His tags for expression in *E. coli* BL21(DE3) as previously described [18]. The expressed antigen represents PIRV VAV-488 N protein residues 296–542 (mw 32 kD).

Recombinant N was diluted in PBS (pH 7.4) and coated onto 96-well polyvinyl chloride plates at 100 ng/well (100 μL) and incubated overnight at 4 °C. Plates were washed 2× with PBS, blocked for 1 h at room temperature with 0.25% gelatin (Sigma G-6144) in PBS and washed 2×. Serum samples (100 μL) were diluted 1:100 in PBS and titrated (log_2_), and incubated for 1 h at room temperature. Wells were washed 3× with PBS-TWEEN-20, followed by incubation of protein-A/G-HRP conjugate (Pierce) for 1 h. After washing 4× with PBS-TWEEN-20, 100 μL of ABTS substrate (KPL) was added for 15 min then absorbance read at 405 nm.

### 2.6. Detection of Viral RNA

Total RNA was extracted from tissues with an RNeasy kit (Qiagen, Hilden, Germany) using stainless steel beads and homogenization (Tissue Lyser II, Qiagen) according to the manufacturer’s instructions. RNA was quantified with a NanoDrop ND-1000 spectrophotometer (Thermo Scientific). One-step polymerase chain reaction (PCR) was performed (Qiagen, 210212) with S segment-specific primers, 5′-CAACCCAGGTTGTGTGTTTG-3′ (forward) and 5′-CAGGTCAAAGGGAGAGCTTG-3′ (reverse), that generated a product of 348 nt.

### 2.7. RNA-Seq

RNA from the livers of two uninfected hamsters (H1, H2) and three diseased hamsters that were euthanized on days 7 (H35, H36) or 8 (H37) were used for transcriptome profiling. RNA-seq libraries were prepared from 500 ng total RNA using Ribo-Zero (Illumina, San Diego, CA, USA) library preparation methods and the manufacturer’s recommended procedure. The five samples were prepared separately and pooled on a single HiSeq 2000 (Illumina) lane for paired end 2 × 100 nt sequencing. Fastq files were quality and adapter-trimmed using the default parameters of Trimmomatic [19]. Using a reference-independent protocol, reads were assembled into contigs using the Trinity package [20] and default parameters. The following options were used: JM 350G, CPU 24, SS_lib_type RF. Within the Trinity package, sample library pairs of 5 samples (2 uninfected and 3 infected) were subjected to RSEM estimation of transcript abundance by aligning them against the Trinity-assembled contigs. Raw count data from RSEM was used as input for DESeq differential expression analysis [21] with a false discovery rate (FDR) *p* value limit of <0.05. DESeq default size factors normalization was applied, as well as the following dispersion estimation parameters: method = “pooled”, sharingMode = “maximum”, fitType = “local” and a minimum of 200 read counts summed across samples. Transcripts were identified by Basic Local Alignment Search Tool (BLAST) against the RefSeqRNA databank (ngKLAST v.r. 4.3, Korilog, http://plealog.com/korilog/). The resulting datafile of differentially expressed genes with transcript counts (Table S1) shows the reference species for each hit. All raw fastq files are available at the National Center for Biotechnology Information (NCBI) Sequence Read Archive under BioProject ID PRJNA258080.

### 2.8. Host Response-Associated Transcripts

Differentially expressed transcripts (DETs) were submitted to InnateDB to identify interferon-associated transcripts [22]. Functional analysis of DETs was performed with the Database for Annotation, Visualization and Integrated Discovery (DAVID) [23] to identify genes relevant to antiviral and host defenses. Candidate lists associated with these responses was generated and submitted for cluster analysis and plotting using R statistical software and the pheatmap plugin. The gene symbol list was analyzed using Reactome [24] and within Cytoscape [25] to construct pathway maps with highlighted DETs.

## 3. Results

### 3.1. Disease Progression

**Figure 1:**
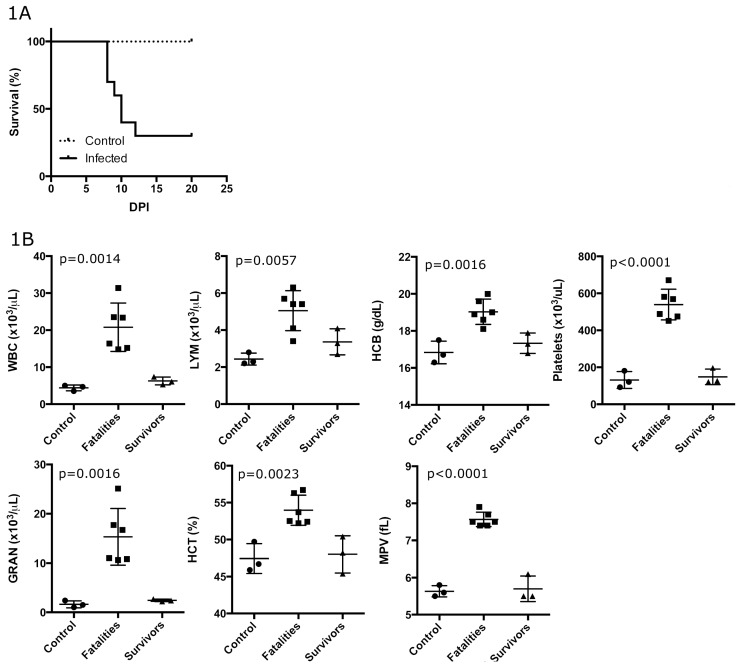

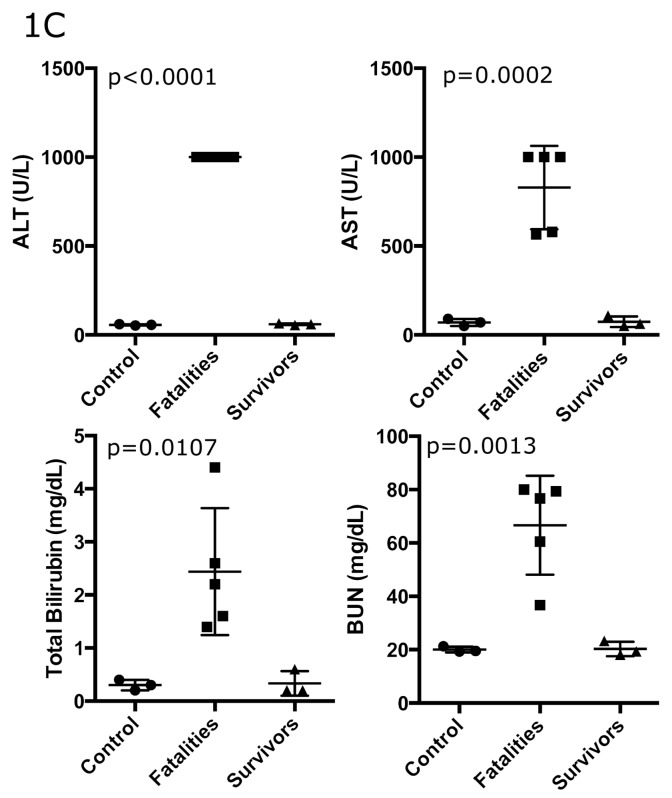
Clinical outcome of Pirital virus (PIRV) infection of Syrian hamsters. (**A**) Seven of the 10 hamsters died by day 12, whereas the remaining 3 infected hamsters appeared healthy until the conclusion of the experiment on day 20. (**B**) Complete blood counts of hamsters showed elevated white blood counts, hematocrit (HCT), hemoglobin (HGB) and mean platelet volume (MVP), and elevated platelet counts. (**C**) Alanine aminotransferase (ALT), aspartate aminotransferase (AST), bilirubin and blood urea nitrogen (BUN) levels were elevated in diseased hamsters but not in infected hamsters that did not show clinical signs of disease.

To confirm previous findings in preparation for differential expression analysis, hematology and histopathology analyses were performed on PIRV IM-inoculated hamsters with clinical signs. Upon development of cutaneous and visceral petechiae with hind-limb paralysis, one hamster was found dead on day 7, five hamsters were euthanized because of severe disease between days 7 and 10, and one euthanized on day 12 because of severe disease; the remaining 3 hamsters showed no signs of disease during the 20-day experiment. Euthanized hamsters exhibited hind-limb paralysis and signs of hemorrhagic disease, including cutaneous and visceral petechiae, and some had significant epistaxis. In total, 7 of 10 inoculated hamsters died or were euthanized (Figure 1A). The hamsters with fatal disease had leukocytosis due primarily to elevated granulocyte and lymphocyte counts, elevated hemoglobin and hematocrit, thrombocytosis, and increased platelet size (Figure 1B). No differences were found in monocyte counts, mean corpuscle volume, red cell distribution width, mean corpuscular hemoglobin concentration, or mean corpuscular hemoglobin. Elevated alanine aminotransferase (ALT), aspartate aminotransferase (AST), total bilirubin and blood urea nitrogen (BUN) levels were also noted (Figure 1C). There were no differences in hematology or blood chemistry between the three exposed, surviving hamsters and negative controls. The three infected but asymptomatic hamsters were antibody positive to nucleocapsid by enzyme-linked immunosorbent assay (ELISA), one mortally-infected hamster was antibody positive (euthanized on day 12), and the remaining six infected hamsters were antibody negative (disease onset on or before day 10) (data not shown). Therefore, RNA-Seq was performed on uninfected negative controls rather than asymptomatic PIRV-exposed animals, and then compared to infected experimental samples.

### 3.2. Histology/Immunohistochemistry

**Figure 2:**
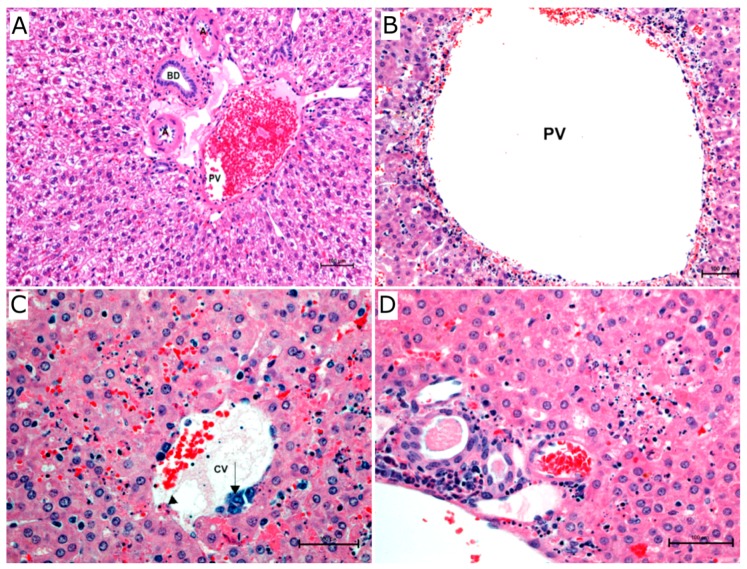
Histopathology of PIRV-infected hamsters. (**A**) Normal liver from a control hamster showing intact limiting plate (hepatocyte layer) surrounding portal triad that contains hepatic artery (A), portal vein (PV) and bile duct (BD). Histopathology of PIRV-infected hamsters 7 days post infection (PI). (**B**) Histologic changes in liver showing circumferential necrosis of the limiting plate with abundant karyorrhectic debris from dead hepatocytes immediately surrounding portal vein (PV) and necrosis and loss of lining endothelial cells (H and E staining, original magnification 100×). (**C**) Liver of infected hamster showing endothelial necrosis (arrowhead) where cells are replaced by eosinophilic cellular debris and pyknotic nuclei with focal intimal hyperplasia of central vein (CV) (arrow) (H and E stain, original magnification 200×). (**D**) Liver of infected hamaster showing random foci of lytic necrosis where hepatocellular details are lost with mixed inflammatory infiltrates expanding portal triads. Bars = 100 μm.

Gross and histopathology analyses were performed to establish a basis for the interpretation of the transcriptomic data. Lesions in infected hamsters were moderately severe-to-marked in the liver and spleen and moderate in the lungs and pancreas of all animals. Hepatic inflammation was typically portal to random and lymphoplasmacytic to neutrophilic in nature. Large areas of hepatic lobules were obliterated by coagulative to lytic necrosis with moderate-to-marked hepatocyte loss. Necrosis of the limiting plate was characterized by punctate or circumferential cell loss (Figure 1A), rounding up of individualized cells with hypereosinophilia of the cytoplasm and pyknosis or lysis of the nuclei. A central vein of one hamster at 7 days post inoculation (PI) showed multifocal intimal hyperplasia (Figure 1B). Reticulin stain revealed partial to complete collapse of most affected hepatic lobules. Immunohistochemistry revealed strong immunoreactivity in scattered Kupffer cells with their characteristic morphology and endothelial cells lining both portal and central veins (Figure 2A).

As for the rest of the other parenchymatous organs, lesions were especially prominent, most consistent in the spleen, lungs and pancreas. Lesions were comprised of organomegaly, multifocal hemorrhages and multifocal areas of pallor or white nodules on gross examination. Splenic lesions in infected hamsters, 7–10 days PI, were moderately severe to marked and were largely characterized by apoptosis and/or necrosis of endothelial cells lining the sinuses of the red pulp. Many of the affected sinuses were filled with fibrin thrombi (sinus thrombosis, Figure 1C). Multifocally, neutrophils and macrophages were degenerate/necrotic along with variably severe lymphocytolysis and subsequent reduction of the overall size of periarteriolar lymphoid

Follicles. Moderate to severe pulmonary lesions were present in four infected hamsters. Multifocal acute interstitial pneumonia was evident in acutely infected animals at 7 days PI, especially in the subpleural parenchyma and around pulmonary vessels. Apoptosis and/or necrosis of pneumocyte type-I along with small foci of necrosis was observed in perivascular lesions. In most affected animals, there was multifocal loss of bronchiolar lining epithelium. In the markedly affected hamsters, inflammation was more of a lymphohistiocytic nature with margination of congested vessels by neutrophils. Pleural mesothelium was multifocally hyperplastic and/or hypertrophic in three hamsters. Pancreatic lesions were moderate to moderately severe. Interstitial edema was multifocally accompanied by segmental loss of exocrine acini. Inflammatory foci were evident in the vicinity of small venules showing apoptosis and/or necrosis of their lining endothelium. In affected vascular intima, endothelial cells were multifocally karyorrhectic and hypereosinophilic.

Consistent immunoreactivity was observed in pulmonary foci of hypertrophic and occasionally hyperplastic pleural mesothelium (Figure 2D). In the lungs, vascular necrosis and perivascular hemorrhage and edema was seen to varying degrees and lumina of affected vessels contained smudged cell debris and chromatin smearing. Small- to medium-sized venules were most affected, followed by capillaries and small arterioles. The wall of these vessels was segmentally to circumferentially obliterated or smudged by leukocytic infiltration into tunica media.

Plasma enzyme levels were more similar among PIRV-infected hamsters and terminal-stage lymphocytic choriomeningitis virus (LCMV-WE) NHP than those reported for the LCMV-infected mouse model. Specifically, ALT but not AST levels were elevated during LCMV-WE infection of inbred lab mice (*Mus musculus*), and neither were elevated in LCMV-ARM infection. The LCMV-WE strain causes disease in non-human primates, whereas the ARM strain is non-pathogenic [26]. In contrast, terminal stage LCMV-WE infection of NHP and PIRV-infected hamsters showed elevated ALT and AST levels, similar to what we observed in PIRV-infected hamsters. Therefore, we chose the LCMV-WE liver differential expression data from the Djavani et al. [16] study rather than LCMV-ARM as the most appropriate comparison for the differential expression data described below.

### 3.3. Differential Expression Profiles

RNA-Seq was performed to gain insight into the substantial liver damage, neutrophil infiltration and inflammation revealed in the histopathology and clinical chemistry. Liver-derived RNA of three fatally-infected hamsters (accession #35, #36 and #37) was compared to two negative controls using differential expression (DE) analysis. A total of 3486 transcripts were differentially expressed, representing 2248 unique genes with known homology (Table S1). Of these, 394 unique genes were predicted to be involved in host response and antiviral-related pathways. DAVID and Reactome analyses revealed that unique enriched immune response transcripts (*n* = 286) had predicted involvement in a variety of innate and adaptive immune responses. Importantly, key hamster immune response transcripts showed expression trends that were coincident with those highlighted in studies of Old World mammarenavirus [16], LASV infection of cynomolgus macaques (*n* = 55), and LCMV infection of rhesus macaques (*n* = 358) (Table S1). In addition, the presence of PIRV S and L segment RNA was detected in RNA-Seq data of analyzed hamsters, and conventional PCR detected vRNA in all diseased hamsters but not the 3 infected hamsters that did not show clinical signs of disease. Specific host-response functional groups are described below.

### 3.4. Hemostasis and Clotting

**Figure 3:**
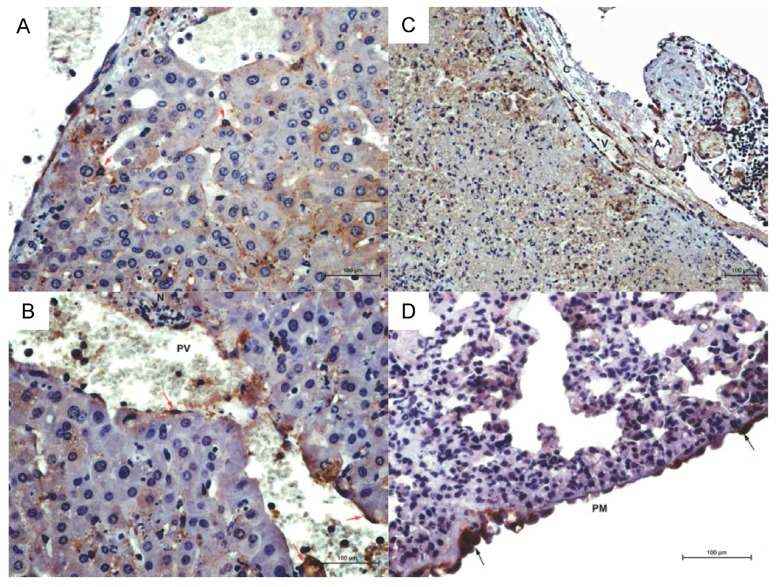
Immunohistochemistry of PIRV-infected hamsters. (**A**) Multifocal immunoreactivity is evident in activated Kupffer cells (red arrows) (original magnification 200×) (**B**) Immunoreactivity is evident in the endothelial lining of PV (red arrows, original magnification). (**C**) Spleen of infected hamster showing strong immunoreactivity of capsular (C) vessels especially veins (V) and to a lesser degree in arteries (A) (original magnification 100×). (**D**) Arrows indicate strong immunoreactivity in hypertrophic and hyperplastic pleural mesothelium (PM) (original magnification 200×). Bar indicates 100 micrometers.

**Figure 4:**
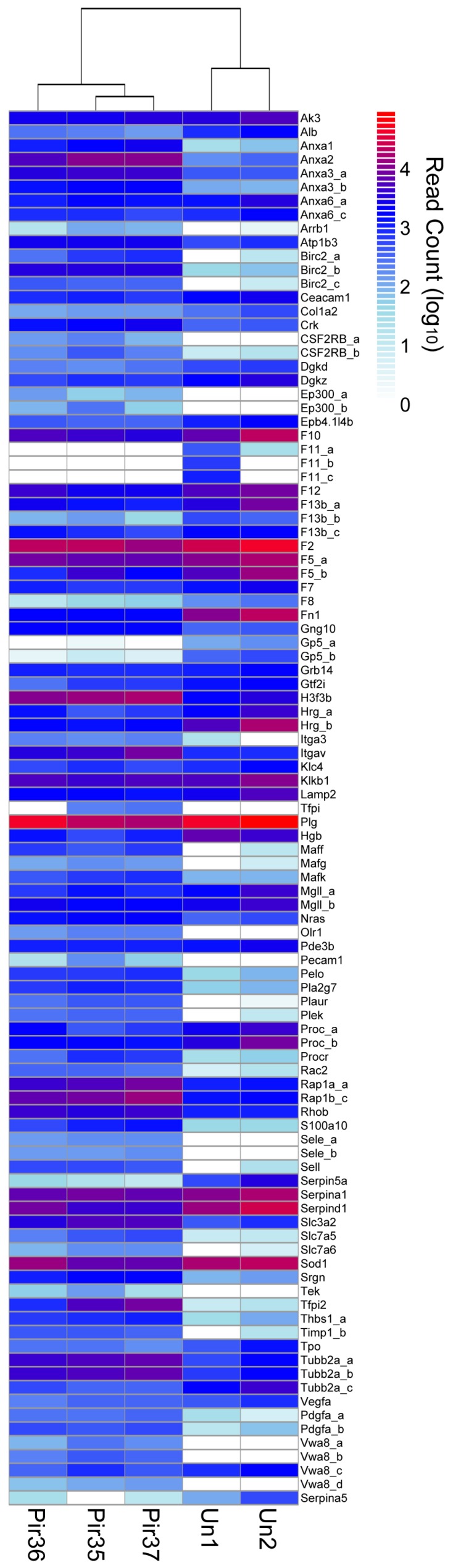
Differentially expressed hemostatic response transcripts (DETs). General innate immune response and cytokine signaling. Differential expression (DESeq) data was plotted by cluster analysis with R software (pheatmap, https://cran.r-project.org). Infected (Pir35, Pir36, Pir37) and uninfected (Un1, Un2) hamster livers. Histogram shows read count densities (log10) for 195 transcript isoforms or alleles from 153 unique genes.

As expected from the hematology (Figure 1) and histopathology findings (Figure 2 and Figure 3), DETs indicative of hematological dysregulation were identified (Figure 4). Importantly, dual enrichment of *Anxa2* and *S100a10* occurred, which has also been reported in the arenavirus NHP model [16]. Annexin A2 and S100 calcium-binding protein A10 form a profibrinolytic complex that promotes plasmin generation [27] and subsequent clot degeneration. In addition, the plasminogen activator urokinase receptor *Plaur* was elevated. Multiple platelet-activating components, including thrombospondin (*Thbs1*), von Willebrand factor A domain (*Vwa8*), platelet-activating factor components *Pafah1b2* and *Pla2g7*, platelet endothelial cell-adhesion molecule (*Pecam1*), and *Serpine1* were elevated. In addition, enrichment of the clotting pathway component DETs, such as tissue-factor pathway inhibitor protein (*Tfpi*), *Cd49b* (alpha 2 integrin) and fibronectin type III domain protein (*Fndc*), was also observed. Enthothelial glycoprotein transcripts *Vcam1*, typically present in cytokine-activated endothelia, and *Icam1*, were not significantly altered; however, they showed a trend toward enrichment in infected liver. Finally, of 79 unique hemostasis-related DETs in PIRV-infected hamster livers, 34 were depleted. These included several clotting factors (*F2*, *F5*, *F7*, *F8*, *F10–F12*). Of these, depletion of *F7*, *F11* and *F12* also occurred in arenavirus-infected NHP [28]. An additional hemostatic regulator, orosomucoid 1 (*Orm1*), showed similar trends in the NHP arenavirus system. Little is known about the function of *Orm1* during arenavirus infection; however, it is classified as an acute phase plasma protein. These results are consistent with the impaired hemostasis that occurs in NHP arenavirus-induced hemorrhagic fevers [16].

### 3.5. Innate Response

**Figure 5:**
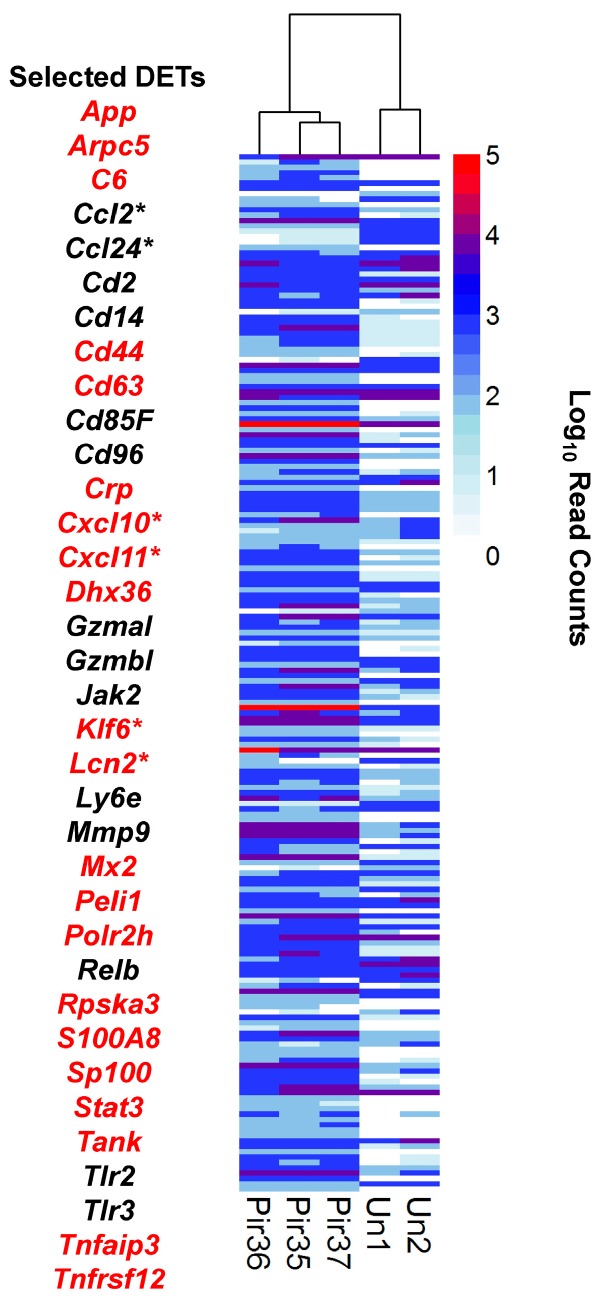
General innate immune response and cytokine signaling. Differential expression (DESeq) data was plotted by cluster analysis with R software (pheatmap). Infected (Pir35, Pir36, Pir37) and uninfected (Un1, Un2) hamster livers. Histogram shows read count densities (log10) for 195 transcript isoforms or alleles from 153 unique genes. Selected transcripts from this set are listed to the left of the histogram; starred transcripts are expected to occur during the pro-inflammatory macrophage M1 response; transcripts in red font are enriched in the liver infected vs. uninfected dataset of Djavani et al. [16].

**Figure 6:**
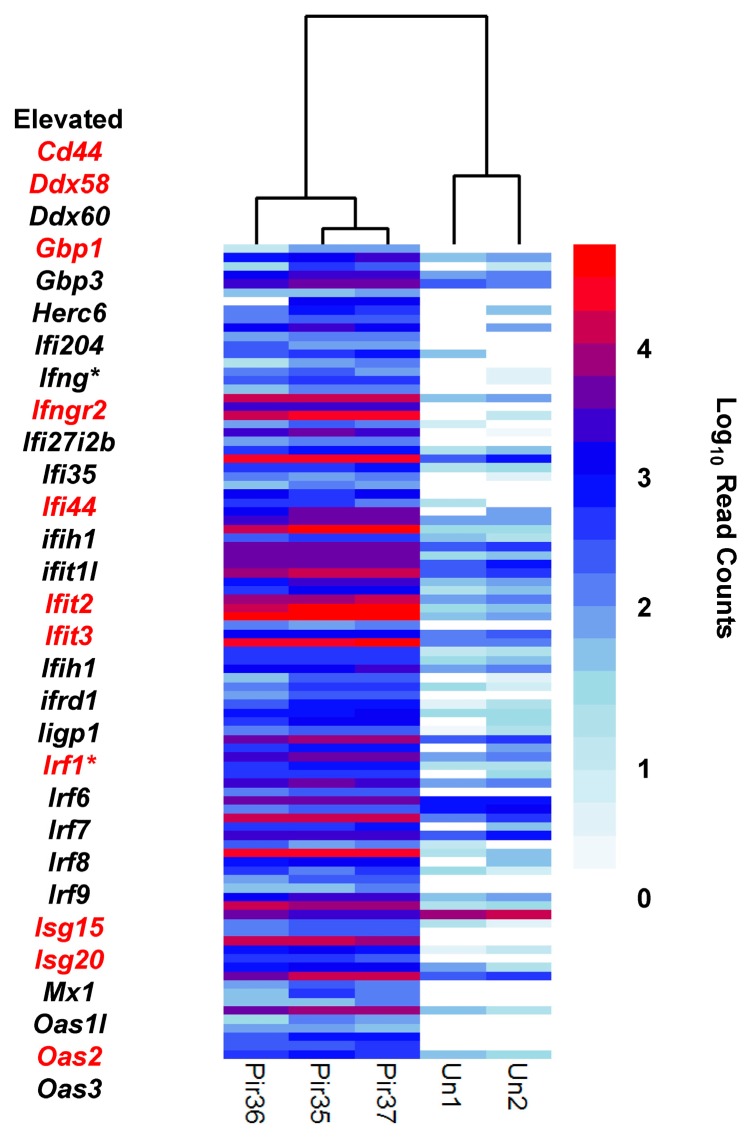
Interferon response. Differential expression (DESeq) data was plotted by cluster analysis with R software (pheatmap). Infected (Pir35, Pir36, Pir37) and uninfected (Un1, Un2) hamster livers. Histogram shows read count densities (log10) for 93 transcript isoforms or alleles from 34 unique genes. Selected transcripts from this set are listed to the left of the histogram; starred transcripts are expected to occur during the pro-inflammatory macrophage M1 response; transcripts in red font are enriched in the infected vs. uninfected liver dataset of Djavani et al. [16].

As expected from other models, innate immune response transcriptional markers were also enriched in PIRV-infected hamster livers (Figure 5 and Figure 6). For example, major components of the cytosolic nucleic acid sensing RIG-I/MDA5 pathway were elevated; DETs included *Ddx58*, *Ddx60*, *Eif2ak2*, the STAT-induced inhibitor *Socs1*, and *Trim32*. Secondly, support for canonical NFκB activation was present in the concomitant transcriptional enrichment of *Nfkb2* and *Relb*, thus favoring NFκB dimer formation. Moreover, ubiquitin antigen-processing pathways also appeared activated, due to enrichment of *Herc6*, *Usp18*, *Trim6 and Tnfaip3*.

The glycosylphosphatidylinositol (GPI) anchor cell-surface protein lymphocyte antigen 6 (*Ly6e*) is a repressor of monocytes, and its enrichment has been associated with increased viral disease progression [29,30]. Importantly, *Ly6e* transcripts were enriched in LASV-infected human peripheral blood mononuclear cells (PBMCs) [31], as well as in other hemorrhagic fevers. Here, PIRV-infected hamster liver also showed enrichment of *Ly6e*, further corroborating the relevance of the hamster pathology system. Lastly, some arenaviruses activate the TLR2 signaling pathway [32,33,34]; similarly, here, *Tlr2* and *Tlr3* expression was elevated in the livers of the infected hamsters.

### 3.6. Interferon Pathway

Transcripts representing type I and III IFNs were not detected, likely because sampling was confined to terminal-stage infections. Nevertheless, several downstream genes of the type I IFN signaling pathway were elevated, consistent with reports of other New World arenavirus human infection [35], including *Ifi27*, *Ifi27l2b*, *Ifi44*, *Ifi204*, *Ifit1*, *Mx1* and *Mx2* (Figure 5 and Figure 6). Likewise, several enriched interferon response components, i.e., *Ifit2*, *Ifit3*, *Ifih1*, *Isg15*, *Isg20* and *Oas2* showed trends consistent with the arenavirus host response in NHP models (Figure 6) [16]. *Zpb1*, a negative regulator of type I IFN signaling [36], was also present and not detected in uninfected samples. These results are consistent with the hypothesis that a type I response had occurred prior to RNA-Seq sampling.

Two genes expected to enhance IFNγ expression (*Tbx21*, *Runx1*) were also elevated, consistent with leukocyte activation (Table S1). Concomitantly, *Ifng* transcripts were present in infected hamster livers but not uninfected controls. Enrichment of *Ifng* is also consistent with the late-stage infection profile for LASV-infected macaque PBMCs. Similarly, the pertinent receptor, *Ifngr2*, was also enriched in our samples and those of LCMV-infected NHP. Moreover, several downstream IFNγR signaling effectors, such as *Stat1*, *Irf7*, *Irf8*, *Gbp1*, *Gbp2*, *Oas2* and *Oas3* were elevated in livers of infected hamsters (Table S1).

### 3.7. Adaptive Immunity

**Figure 7:**
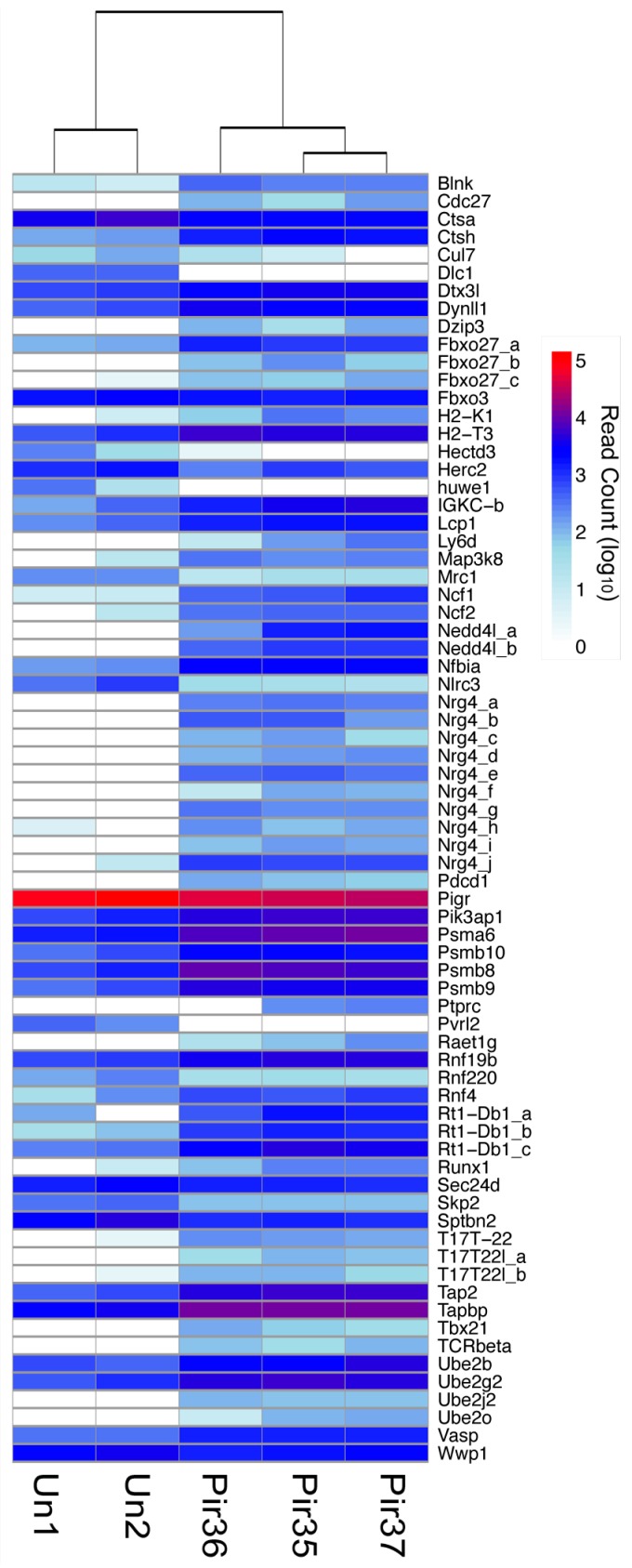
Differential expression of adaptive immunity transcripts. Differential expression (DESeq) data was plotted by cluster analysis with R software (pheatmap). Infected (Pir35, Pir36, Pir37) and uninfected (Un1, Un2) hamster livers, as described in Figure 6.

Many adaptive immunity components associated with MHC class I antigen processing and presentation were also identified (Figure 7). Of 43 unique DETs in this category, 30 were elevated, suggesting many components of the antigen-processing pathway were functional. Of these, about one third were similarly enriched in the Djavani et al. study (*n* = 13) and one (*Psmb8*) in the Malhotra et al. study [16]. T-cell receptor αβ and Igκ sequences were also elevated in the livers of infected hamsters, which is consistent with the observed lymphocyte count elevation (Figure 1).

Several DETs associated with adaptive immunity were depleted, including a cluster of five genes (*Cul7*, *Fbxo3*, *Huwe1*, *Rnf220*, *Skp2*) involved in the cytosolic polyubiquitination of polypeptides immediately prior to proteasomal processing and loading onto nascent MHC class I proteins. Likewise, IgA/IgM epithelial transport receptor (*Pigr*) transcripts were depleted, suggesting that export of these classes to the luminal epithelial surface of the liver may have been impaired. *Cd3* sequences were also detected, as were transcripts for *Cd49b*, which may have been expressed from natural killer (NK) or natural killer T (NKT) cells [37]. Both *Lag3* and *Cd226* transcripts were also elevated in infected livers. Along with *Cd49b*, these are defining markers of T regulatory 1 (Tr1) cells [38]. In addition, *Lgals9*, which encodes Galectin-9, was significantly elevated in livers of infected hamsters. Galectin-9 drives development of regulatory T cells that can attenuate antiviral T-cell responses [39].

### 3.8. Mononuclear Phagocyte System

The mononuclear phagocyte system (MPS) is a multi-cellular network made up of monocytes, macrophages and dendritic cells (DCs). Expression of *Ifng* and histopathological evidence of immunoreactive Kupffer cells and extracellular damage, suggestive of activation of the pro-inflammatory arm of the MPS, led us to assess transcript markers of MPS activation within the PIRV-infected liver RNA-Seq dataset. Extensive evidence of macrophage activation was identified, but no markers for DCs or monocytes were apparent (as reviewed in [40]). Specifically, no transcripts indicative of Batf3-dependent classical DC1 (cDCs) differential expression were present, nor were Irf-4 dependent cDC2 lineage markers, with the exception of *Cd11b/Cd18* (Table S1). It has been argued that Cd11b is more accurately assigned to macrophages and monocytes rather than cDCs [41]. Moreover, there were no differentially expressed markers of monocytes (*iNOS*, *Cd16*, *Cd1c*). This is consistent with the lack of difference in monocyte counts between infected and un-infected controls. However, there was extensive evidence of macrophage lineage cells, consistent with a bias toward a pro-inflammatory response.

Differentially expressed macrophage markers were delineated as M1, pro-inflammatory or M2, pro-Th2 (Table S1). Figure 5 and Figure 6 show highlighted pro-inflammatory markers. For example, plasminogen activator urokinase receptor *Plaur* was elevated. Two additional MPS markers [42], which also physically interact in vivo and contribute to inflammation, lipocalin 2 (*Lcn2*, Log_2_ FC 6.0–6.4, FDR *p* = 5.8 × 10^−4^) and matrix metallopeptidase 9 (*Mmp9*, present in infected, FDR *p* = 4.4 × 10^−3^) were also elevated (Figure 5, Table S1). Further, *Il1b* and several associated transcripts, including transcripts for IL-1 receptors, accessory proteins and antagonists, were enriched, which was consistent with acute inflammation (reviewed in [43]). Chemokine-signaling ligand transcripts C*xcl10* and C*xcl11*, expected to be expressed from activated macrophages, were also elevated [41]. Moreover, *Klf6* (Kruppel-like transcription factor) transcripts were enriched [44].

Of 17 differentially expressed macrophage markers in the dataset, 16 were consistent with a pro-inflammatory M1-type response, and just 1 DET was representative of an M2 healing or cellular remodeling response (Table S1) [43]. Depletion of the transcript encoding the vascular endothelial growth factor, *Vegfa*, was consistent with M2 extracellular matrix remodeling.

### 3.9. Apoptosis and Autophagy Pathways

Many genes involved in ubiquitination were differentially expressed, most of which were elevated in infected hamsters (Table S1). *Isg15*, *Mdm2*, *Siah1*, *Ubd* and *Usp18* were substantially elevated, whereas *Hectd3*, *Huwe1* and *Usp32* were depleted. In addition, transcripts of genes associated with apoptosis were significantly elevated, including *Aen*, *Birc3*, *Cflar*, *Coxc6a2*, *Fas*, *Fbf1*, *Ivns1abp* and *Serpinb10* (Table S1), consistent with previously reported JUNV-induced apoptosis [45].

## 4. Discussion

Syrian hamsters infected with PIRV develop clinical disease that shares similarities to Lassa and LCMV-WE hemorrhagic fevers [16]. Indeed, 363 unique transcripts had coordinated trends among PIRV-infected hamsters and LCMV-infected NHP [16]. Of these, 17 hamster DETs were also common to Lassa virus-infected NHP liver transcriptome signatures (18). These included hallmarks of the innate immune response, such as *Ddx58* (RIG-I), *Oas2*, several additional interferon response genes (*Ifi44*, *Ifit2*, *Isg15*, *Mx2*) and the general lymphocyte proliferative marker, *Cd69*. Therefore, compared to NHP models, the overall hamster PIRV transcriptome signature was more similar to the associated New World representative LCMV than Old World LASV infection.

The innate immune response/IFN category (*n* = 32) contained the highest number of coordinately regulated transcripts among the hamster and LCMV NHP model systems (Table S1). Furthermore, dysfunction of clotting cascades, leading to thromobosis and/or hemorrhage, is a principal feature of Argentine hemorrhagic fever. Although the mechanisms that govern this are unknown, inhibition of aggregation may disrupt platelet function. Interestingly, 22 unique hemostasis transcripts were common among PIRV and LCMV-WE liver infections [16]. Therefore, although there are likely differences among rodents and NHP in antigen-presenting components, important hemostatic and innate immune response markers are similar. Conspicuously, IFNβ sequences were absent in the assembled transcriptome. A BLAST search of the Syrian golden hamster using the mouse IFNβ sequence returned no hits. An annotated hamster genome is available and has 115× coverage (BioProject PRJNA77669), suggesting IFNβ may be absent in hamsters. This may be a consequence of the highly inbred nature of the Syrian golden hamster used in biomedical research, which was founded with one male and two females that were littermates (see S. Alder, 1948, Nature 162:256), and which may explain why Syrian hamsters are highly susceptible to a number of viruses.

The M1 pro-inflammatory arm of the MPS may contribute to pathogenesis of acute PIRV infection in hamsters, which is similar to trends observed in LCMV infection of NHP (17). The spleen, liver and lungs are the largest units of the MPS which, in part, explains why these organs are the most consistently involved targets of PIRV infection. Strong immunoreactivity of the Kupffer cells and endothelium of hepatic sinusoids and central veins corroborates findings in previous studies of PIRV infection in hamsters and hepatotropism [15]. In the absence of significant macrophage infiltrations around foci of hepatic necrosis, the current study corroborates the central role that Kupffer cells play in the progression of PIRV infection. Moreover, the strong immunoreactivity detected in the cytoplasm of mononuclear cells lining hepatic sinusoids is further evidence as to the role of fixed macrophages in Pirital virus-induced pathology. The morphology of these cells is unmistakable. Consistent elevation of coagulation values in PIRV-infected hamsters [15] and sinus splenic thrombosis, reported here, are consistent with the presence of procoagulant molecules. Pirital infection of the MPS is a characteristic reminiscent of virus-induced fulminant hepatitis [46,47]. In addition to sinusoidal coagulation, which hampered effective circulation in the liver contributing to widespread necrosis of hepatic parenchyma, destruction of the limiting plate and loss of the supporting reticulin network reduced the ability of the liver to regenerate. Similar pro-inflammatory signatures were present in LCMV-infected NHP livers (17). For example, enrichment of *Il6r*, *Il1r*, and evidence of TNF (*Tnfrs12*, *Tnfrs1b*) and cytokine activity (enrichment of *Il6R*/*Il1R*) were consistent with a pro-inflammatory M1 response (42). Additional pro-inflammatory markers in the NHP liver dataset included enriched *Klf6*, *Stat1* and *Irf1*, as well as *Il23* in late-stage infection.

Two biomarkers for arenavirus hemorrhagic fevers were identified in the transcriptome data. The first was *Lcn2*; because the protein is excreted in the urine, it may have diagnostic value for assessment of the prognosis of hemorrhagic diseases, similar to its use in human liver and renal disease [48]. Importantly, *Lcn2*, which as a cell-surface protein transports iron, was also among the DETs common to PIRV-infected hamster liver and arenavirus NHP liver pathology models. The predicted function of *Lcn2* differs depending on whether it is cell-surface bound or secreted. As a cell-surface molecule, it internalizes iron. Alternatively, the matrix metalloprotease Mmp9 and *Lcn2* form a heterodimer which, when secreted from neutrophils and macrophages, promotes extracellular remodeling [49]. Similarly, the secreted plasminogen activator, urokinase receptor (*Plaur*), is expressed from macrophages [50]; moreover, the presence of soluble *Plaur* in patient plasma has been suggested as a diagnostic tool for Crimean-Congo hemorrhagic virus infection [51] and could be used for arenavirus disease in a similar manner. This transcript was dramatically enriched in PIRV-infected livers (Log_2_ FC 8.2, FDR *p* = 7.2 × 10^−7^, Table S1). Although we did not specifically assay circulating markers, elevation of *Plaur* and *Lcn2* transcripts and co-implication of M1 pro-inflammatory involvement are consistent with the hypothesis that the associated gene products could be used as indicator markers.

Aside from those common features, the following signatures were noted in the PIRV-infected liver dataset. Two genes, *Pafah1b2* and *Pla2g7*, significantly elevated in PIRV infected hamsters, encode inhibitors of the platelet activating factor (*Paf*) that cleave it into an inactive form [52,53]; their expression may contribute to failure of platelet aggregation and explain, in part, the vascular leakage and hemorrhage observed in arenavirus disease. Two additional genes, which result in inhibition of the clotting cascade, *Tfpi* and *Tfpi2,* may have similar effects. Several other transcripts (*Thbs1*, *Plaur*, *Serpine1*) involved in coagulation were elevated in liver tissue and these genes likely have a participatory role in the observed coagualopathy that occurs during PIRV infection. In addition, a transcript for a *Vwf* accessory protein, von Willebrand factor A domain containing 8 (*Vwa8*), was elevated in infected livers. The antiviral response of hamsters infected with PIRV showed substantial signs of innate, interferon and ubiquitination pathway activation. Early events included genes associated with pattern recognition receptor (PRR) sensing and signaling, including RLH, MDA and ubiquitin pathway mobilization. *Tlr2* expression was higher in infected hamster livers (Log_2_ FC 6.1, FDR *p* = 3.0 × 10^−7^) and *Tlr3* transcripts were present in all infected hamsters but absent in uninfected. The TLR-2 pathway is targeted by pathogenic arenaviruses [34,54], therefore enrichment of *Tlr2* is consistent with other reports. TLR-3 is not involved in cytotoxic T lymphocyte (CTL) mediated responses to LCMV infections [55] and its elevated expression in PIRV infected hamsters may indicate a viral and/or host species difference in responses. Further investigation will be required to address this.

Several transcripts associated with NK cell subsets and CTL activities were differentially expressed. Some of these genes are shared by NK and T cells (e.g., *Ifng*, *Cd3d*, *Cd3e*, *Cd3g*, *Cd49b*, *Perf*, *Gzma*, *Gzmb*). T-cell receptor α and β gene sequences were elevated in infected hamsters livers, suggesting T-cell participation in the host response. In LCMV infection of laboratory house mice, T-cell responses occur early and, depending on the strain of virus and mouse, have different outcomes, including disease and clearance, or persistent infection [56]. Some LCMV clones induce immunosuppression [57,58,59] or exhaustion of responding CTLs and these events are controlled by cell-surface interactions between CTLs and other cells. Although *Cd8a* and *Cd8b* transcript counts were below our threshold cutoff of 200, they were substantially higher in infected hamster livers than in uninfected livers, whereas *Cd4* transcripts were absent in all samples (Table S1). Together with the elevated TCR*ab* transcript data, this suggested CD8^+^ CTL infiltration of the livers in response to infection but that these cells were likely ineffectual. An important feature associated with T-cell exhaustion is PD-1-induced immunosuppression of CTLs, an event mediated by PD-1 ligands (PD-L) found on other cells [60,61,62]. *Pdcd1*, which encodes PD-1, was present in infected hamsters but absent in uninfected livers (Table S1). In addition, transcripts associated with regulatory T cells (*Cd226*, *Cd49b*, *Lgals9*, *Lag3*) were elevated, suggesting another negative impact on CTL activity. Genes expressed during MHC class I antigen processing were highly elevated and we did not detect repression of any genes associated with this pathway, suggesting it was functioning as expected. Future experiments could examine the role of PD-1/PD-L by use of blocking antibodies that could restore functional CTL activities. It is unclear if such antibodies exist for use in hamsters; however, several mouse- and rat-specific monoclonal antibodies have been identified that are cross-reactive with hamster cells [63], and thus it may be that cross-reactive PD-1/PD-L antibodies are already available for such studies in hamsters.

Of particular interest are the hamsters that survived infection. Syrian hamsters have limited genetic homogeneity, and thus an explanation for why 30% survived without conspicuous signs of disease could provide important clues about resistance, may point to novel approaches for mitigating disease, and could provide prognostic indicators of infectious outcomes. The development of non-lethal methods that provide meaningful data for serial sampling in hamsters will be required for future studies in order to identify changes associated with disease progression and resistance.

## 5. Conclusions

Collectively, the data presented here are consistent with a robust antiviral innate immune response, including signatures of pro-inflammatory M1 macrophage response, in Syrian hamsters infected with PIRV, with concomitant CTL exhaustion and/or regulatory T-cell responses that may impair the transition to adaptive immunity for successful control of the infection.

## Supplementary Materials

Supplementary materials can be found at http://www.mdpi.com/1999-4915/10/5/232/s1. Table S1: Differential gene expression profiles of Syrian hamsters infected with PIRV.
